# Larval habitat diversity and mosquito species distribution along the coast of Kenya

**DOI:** 10.12688/wellcomeopenres.15550.1

**Published:** 2019-11-13

**Authors:** Miriam Karuitha, Joel Bargul, Joel Lutomiah, Simon Muriu, Joseph Nzovu, Rosemary Sang, Joseph Mwangangi, Charles Mbogo

**Affiliations:** 1 Vector Biology Unit, Kenya Medical Research Institute (KEMRI), Center for Geographic Medicine Research Coast, Kilifi, P.O. Box 230-80100, Kenya; 2 Department of Biochemistry, Jomo Kenyatta University of Agriculture and Technology, Juja, Kenya; 3 The Animal Health Department, International Centre of Insect Physiology and Ecology, Nairobi, P.O. Box 30772-00100, Kenya; 4 Hemorrhagic Fever Unit, Kenya Medical Research Institute (KEMRI), Center for Virus Research, Nairobi, P.O. Box 62000-00200, Kenya; 5 Department of Biological Sciences, Pwani University Bioscience Centre (PUBREC), Kilifi, P.O Box 230-80100, Kenya; 6 Kenya Medical Research Institute (KEMRI), Center for Vector Disease Control, Kwale, Kenya; 7 KEMRI-Wellcome Trust Research Programme, Nairobi, P.O. Box 43640-00100, Kenya

**Keywords:** Larval habitats, habitat productivity, Aedes, Culex, culicine diversity, Arbovirus

## Abstract

**Background:** Management of arboviruses relies heavily on vector control. Implementation and sustenance of effective control measures requires regular surveillance of mosquito occurrences, species abundance and distribution. The current study evaluated larval habitat diversity and productivity, mosquito species diversity and distribution in selected sites along the coast of Kenya.

**Methods:** A cross-sectional survey of mosquito breeding habitats, species diversity and distribution was conducted in urban, peri-urban and forested ecological zones in Mombasa and Kilifi counties.

**Results:** A total of 13,009 immature mosquitoes were collected from 17 diverse aquatic habitats along the coast of Kenya. Larval productivity differed significantly (F _(16, 243)_ = 3.21, P < 0.0001) among the aquatic habitats, with tyre habitats recording the highest larval population. *Culex pipiens *(50.17%) and *Aedes aegypti* (38.73%) were the dominant mosquito species in urban areas, while *Ae. vittatus* (89%) was the dominant species in forested areas.  In total, 4,735 adult mosquitoes belonging to 19 species were collected in Haller Park, Bamburi, Gede and Arabuko Sokoke forest. Urban areas supported higher densities of *Ae. aegypti* compared to peri-urban and forest areas, which, on the other hand, supported greater mosquito species diversity.

**Conclusions:** High *Ae. aegypti* production in urban and peri-urban areas present a greater risk of arbovirus outbreaks. Targeting productive habitats of *Aedes aegypti*, such as discarded tyres, containers and poorly maintained drainage systems in urban areas and preventing human-vector contact in peri-urban and forested areas could have a significant impact on the prevalence of arboviruses along the coast of Kenya, forestalling the periodic outbreaks experienced in the region.

## Introduction

Different mosquito species serve as vectors of human pathogens including, yellow fever virus (YFV), chikungunya virus (CHIKV), Zika virus (ZIKV), dengue virus (DENV), Rift Valley fever virus (RVFV), West Nile virus (WNV), O’nyong-nyong (ONNV) and those that cause malaria and lymphatic filariasis, mainly in tropical and sub-tropical regions ^1– 9^. Among the three mosquito subfamilies of Toxorhynchitinae, Anophelinae and Culicinae, only the anophelines and culicines have been incriminated in human pathogen transmission. The anophelines are largely important in the transmission of malaria parasites, filarial nematodes and arboviruses. Culicine mosquitoes have been implicated in the transmission of a wide range of arboviruses, with species in the *Culex* and *Aedes* genera playing a key role ^2, 3, 5, 9– 11^.

The genus *Aedes* has been shown to transmit the majority of arboviruses including YFV, DENV, RVFV, CHIKV and ZIKV in both endemic and epidemic outbreaks ^3, 5, 6, 9, 10, 12, 13^. The sylvatic transmission cycle of the majority of these arboviruses mainly involves *Ae. africanus, Ae. furcifer, Ae. luteocephalus, Ae. keniensis Ae. bromeliae, Ae. hirssutus, Ma.uniformis* and *Ae. hensilli* ^4– 6, 10, 14^. *Ae. aegypti* is considered to be the key epidemic vector of YFV, DENV, CHIKV, and ZIKV. Other vectors of arboviruses include *Anopheles* species that transmit ONNV and *Cx. pipiens* and *Cx. Univittatus*, which are vectors of WNV in Africa ^2, 10, 14– 16^. Transmission of RVFV involves an array of mosquito species such as *Ae. mcintoshi*, *Ae. ochraceus*, *Mansonia*, *Cx. quinquefasciatus* and *Cx. annulioris* ^7, 9^.

Kenya has a history of different arboviruses outbreaks including YFV, RVFV, DENV and CHIKV in different parts of the country ^17– 22^. Several cases of dengue fever outbreak were reported in 2013 and 2014 in Mombasa and its environs, where more than 100 cases of infection with dengue fever were confirmed. The majority of the infected patients were the elderly and children ^13, 19, 23^. Recent outbreaks of dengue and chikungunya were reported in Mombasa along the coast of Kenya and Mandera in north eastern Kenya in 2017 and 2018 ^24– 26^.

Mosquito species diversity varies with ecological and environmental conditions, with some species present in cold/temperate regions and others in dry environments ^2^. The Kenyan coast is characterized by high temperatures ranging between 24–33°C and an average relative humidity of 80%, which are optimal conditions for breeding for most mosquito species that transmit malaria, arboviruses and filarial worms. In addition, different habitats suitable for different species are readily available although poorly characterised.

Culicine mosquitoes are known to breed in diverse habitats and occur in different environments, some species of which have adapted to colonise urban centres. For instance, *Cx. quinquefasciatus (*a member of the *Cx. pipiens* complex), a vector of filarial worm, WNV and a secondary vector of RVFV, breeds in organic polluted water in cess pits, drainage canals, and sewerage systems ^1, 2, 27, 28^, while *Ae. aegypti* prefers shallow water mostly collected in tyres, plant axils, household utensils and other containers readily available in urban cities with poor garbage management ^2, 13, 29^.

The rate of vector-borne disease transmission depends on vector abundance and distribution, the presence of diverse larval habitats and human lifestyle ^30^. Mosquito larvae are highly restricted to their habitats with minimal chances of evading control measures as compared to free-flying adult mosquitoes, which makes larviciding an effective control strategy. Integrating larval source management (LSM) with adult control methods significantly reduces mosquito populations ^29, 31– 33^. Adult and larval surveillance plays an important role in the provision of information on mosquito species and habitat distribution for the design of effective control strategies.

 The current study was conducted to establish mosquito species diversity, breeding habitats and their distribution in selected sites within Mombasa and Kilifi Counties so as to provide information that could contribute to effective and successful control of arbovirus vectors.

## Methods

### Ethical considerations

Ethical approval was obtained from the Scientific and Ethical Review Unit (SERU) of Kenya Medical Research Institute (KEMRI/SERU/CVR/04/3442). Consent to carry out sampling within the forest ecosystem was sought from Kenya Wildlife Services and Haller Park management prior to commencement of the study.

### Study area description and site selection

**Figure 1.**
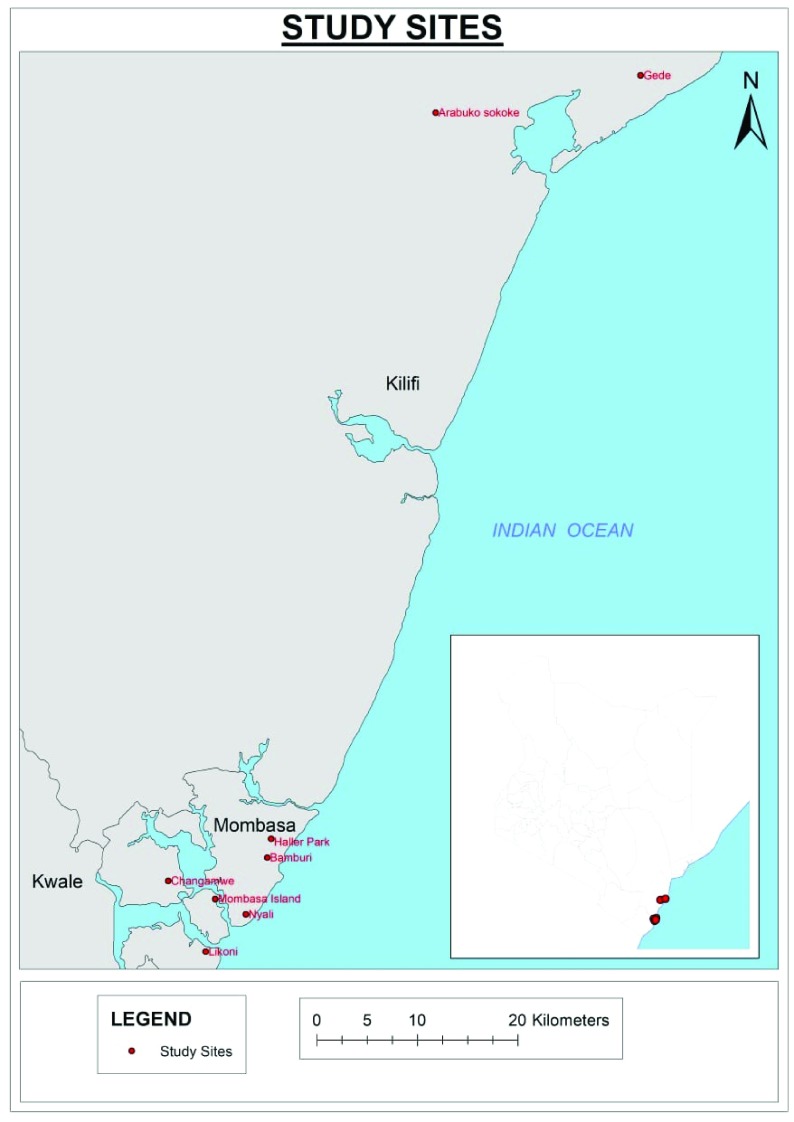
Map showing the study sites along the Kenyan coast where the surveillance of breeding sites and mosquito collection was conducted.

The study was conducted in two forested areas (Arabuko sokoke forest and Haller Park) and two peri-urban (Gede and Bamburi) areas within Kilifi County and in Mombasa County and urban areas within Mombasa Island and its environs along the Kenyan Coast, as shown in Figure 1. Mombasa Island lies at 4°0’S latitude and 39°4’E longitude and the land within this area is mainly used for commercial and residential purposes. Other areas studied in Mombasa included Haller Park (4°1′0″S, 39°43′10″E), a nature trail formerly known as Bamburi nature trail located 12km north of Mombasa city along the Mombasa-Malindi highway and south of Bamburi Cement Plant. The Park is a product of Dr. Rene Haller, who wished to rehabilitate the abandoned and forgotten limestone quarries to the current lucrative tourist attraction trail along the Kenyan coast. It hosts a variety of wildlife including buffalos, giraffes, hippos, waterbuck, eland, oryx, monkeys (green vervet monkey, Sykes’ monkey and mona monkey) and antelopes. It is also a home to over 160 species of birds, weaver birds (Taveta golden weaver, black-headed weaver and golden palm weaver), porcupines, Aldabra tortoises, snakes and crocodiles among others. These animals and birds were introduced from diverse ecosystems ^34– 38^. Bamburi (4°0′S, 39°43′E) is a commercial, industrial and residential peri-urban area in Kisauni sub-county on the north of Mombasa City. The area is inhabited by middle- and low-income earners and is home to several tourist sites including Jomo Kenyatta Public beach, Haller Park and Hotels. Nyali (4°3′0″S, 39°42′0″E) is a prime residential mainland area of Mombasa, accessible from Mombasa Island by road via the Nyali bridge. Likoni (4°5′0″S, 39°39′0″E), a mainland on the south of Mombasa City is accessible only by ferry through Likoni creek, linking Mombasa to the south coast and is mainly inhabited by low-income earners. Changamwe (4°1′34″S, 39°37′50″E) is an industrial mainland suburb west of Mombasa Island, accessible by foot, road or rail through Makupa Causeway.

Arabuko-Sokoke forest (3°35′81′61′′S, 30°89′90′82′′E) is a protected national forest reserve in Kilifi County, 110 km north of Mombasa City, is approximately 370 km ^2^ in size and is managed by the Kenya Forest Service (KFS). It is the largest intact coastal forest in East Africa and hosts 52 mammal species including elephants, buffalo, civet, yellow baboons, lesser galago, among others, and wide range of vegetation ^39, 40^. Gede (3°28′0″S, 39°18′0″E) is a peri-urban area along the Mombasa-Malindi highway. Gede is headquarters to Kenya Forest Research Institute (KEFRI), Gede Centre and Gede Ruins National Monument and Museum, an old Swahili tourist attraction and nature trail. The majority of the residents are locals and Kenya Wildlife Services (KWS) rangers.

Mombasa Island and its neighbouring mainland towns, Likoni, Changamwe and Kisauni, were surveyed for mosquito breeding sites in 2014. Haller Park, Arabuko-Sokoke forest, Gede and Bamburi were surveyed in 2016 for adult species diversity, mosquito eggs as well as breeding sites. In general, coastal Kenya experiences two rainy seasons; long rains (April-June) and short rains (November-December), with a total annual precipitation of 1192mm, and average annual temperature and humidity in the region is 24.7°C and 80%, respectively. The driest season is experienced between January and February. The site selection for larval surveillance was based on 2013/2014 confirmed dengue cases in Mombasa Island and its neighbouring mainland towns ^13^. Others were selected based on the role they play as tourist attraction sites and their proximity to Mombasa City. There is no information available on mosquito species diversity in Haller Park and the significant role they play as arboviruses reservoir in a sylvatic cycle. On the other hand, mosquito vectors in the two peri-urban areas, Bamburi and Gede, neighbouring Haller Park and Arabuko-Sokoke (forested areas), respectively, could play important role in initiating urban cycle, hence the outbreak. Thus, adequate surveillance of the mosquito species distribution and diversity is necessary for planning and implementation of effective vector control.

### Collection of mosquito eggs

*Aedes* mosquito eggs were collected using black disposable plastic glass ovicups placed in randomly selected potential oviposition sites, at least 100m apart at places that could hold water during the rains, such as rock holes, between branches, under shrubs and between bamboo trees, in Haller Park and Arabuko-Sokoke forest. At each sampling site, the labelled ovicups were fitted with filter paper, half filled with water, secured on the site and retrieved five days later. The filter papers lining the ovicups were air dried, packed in individually labelled A7 white envelopes and transported to a biosafety level 2 insectary at Kenya Medical Research Institute-Centre for Virus Research (KEMRI-CVR). The dried eggs were dispensed in larval trays to hatch and the larvae reared to adults under controlled laboratory conditions of 28°C and 70% humidity. The emerging adults were knocked down by placing in small cages at 4°C for 5 minutes and preserved at -80°C in 1.5ml cryogenic tubes for further processing.

### Larval habitat identification and characterization

Mosquito larval sampling was conducted between August and October 2014 in Mombasa Island, Likoni, Changamwe and Kisauni and from November to December 2016 in Haller Park, Arabuko-Sokoke forest, Gede and Bamburi. Habitat characteristics such as water depth, type of breeding habitat, habitat size, permanency, amount of vegetation cover, amount of shade, age of the habitat, substrate type, presence of predators, water flow and water colour were recorded. Breeding habitat was defined as either completely, partially or not shaded by any urban structures or nearby foliage. Permanency was determined by the presence or absence of constant water source; the habitats without constant water supply were considered temporary due to their likelihood to dry up. Vegetation cover was defined as none, some or many plants/grasses around the breeding habitat. Amount of shade was defined as shaded if the habitat had limited access to sunlight and partially shaded if the habitat was not completely shielded from direct sunlight. The age of the habitat was scaled from less than one month to over one year and was based on the information provided by public health officers working in the areas of study. Habitat substrate was defined as breeding habitats with mud, sand, gravel or artificial substrates. The presence of predators was assessed by identifying whether tadpoles, fish or other insects, such as dragonflies, that feed on mosquito larvae were present in the habitats. Water flow was defined as fast flowing, slow flowing or stagnant water and colour defined as clear, black, brown or green, classified based on its appearance by eye. Containers were defined as any water-holding item sampled with a volume between 0.5L to 50L ranging from jerry cans, plastic buckets, plastic and metal drums, plastic basins, plastic water bottles and blue band containers.

Depending on the habitat size, mosquito larvae were sampled using either a standard dipping technique, where at least three dips were taken at different points within each habitat using a standard 350ml dipper, or pipetting techniques, where all the water in small breeding habitats was emptied onto white larval rearing trays and all the larvae present picked using a 1ml pipette. One to three dipper samples were taken along the habitat edge depending on the habitat size using a 350ml dipper ^41^. In small habitats where the 350ml dipper could not be used or where the site contained less than half a litre of water, a 1ml transfer pipette was used to collect mosquito larvae and pupae. The samples for each habitat at each sampling site were transferred onto a white larval rearing tray, enumerated by picking individual larvae with a pipette, pooled into a Whirl-Pak and transferred to the laboratory in a cool box for rearing, identification and further processing.

### Adult mosquito collection

Adult mosquitoes were collected from two study sites in Mombasa County (Haller Park and Bamburi), and two in Kilifi County (Arabuko-Sokoke forest and Gede) between November and December 2016. The adult mosquitos were collected with the use of a BG-Sentinel trap (Biogents), CO _2_-baited CDC light trap or CDC resting trap. In each of the sampling locations, ten sets of BG-Sentinel and light traps and five sets of resting traps were set randomly at different points within the same study area at 1800 hours, away from any visible animal or human paths, and collected between 0600 and 0800 hours the following day. Another set of traps were placed at different locations within the same site at 0600 hours, targeting diurnal feeding mosquitoes, and retrieved at 1800 hours. Trapped mosquitoes were knocked down by placing a paper towel soaked in triethylamine acetate (TEA) in a clear polythene bag containing the adult mosquito traps for three minutes to immobilize the adult mosquitoes, then sorted to remove non-targeted insects, and preserved in liquid nitrogen shipping vessels for transportation to KEMRI-CVR in Nairobi for identification and further processing.

### Laboratory processing

The larvae from each aquatic habitat were transferred into white enamel trays for rearing at the insectary. The date of collection, habitat type and site were labelled. The pupae were placed in pupae cages and reared to adults in an insectary at 28°C and 70% humidity. Adult mosquitoes were identified morphologically under a microscope on a cold plate to species level using identification keys described by Jupp *et al.*, Edwards *et al.*, Harbach *et al.*, Gillet *et al.* and Gillies *et al.* ^42– 46^, and pooled into groups of up to 25 mosquitoes in each 1.5 ml Eppendorf tube according to species, sex, site, and collection date and frozen at -80°C for future processing.

### Data analysis

Data were entered in Microsoft Excel and analysis conducted using STATA software (version 12 for windows). Overall survivorship of adults emerging from egg collection was estimated by dividing the total number of adults (A) by the total number of first instar larvae that hatched (L1) ^47^. The distribution of mosquitoes in the study area was analysed by calculating the abundance as the ratio of mosquito species population per site to the total number of mosquitoes collected in that site. One-way analysis of variance (ANOVA) test was used to analyse the variation in *Culex* and *Aedes* larvae production from different habitat types. Larval density was analysed by dividing total number of larvae per habitat by number of dippers collected. Pipette collection per habitat was assumed to be one dipper for the analysis. Larval density was log transformed, log _10_ (x+1), to normalize the distribution. Linear regressions were used to test the relationship between the culicine larval population and environmental variables. Shannon diversity and evenness indices (H) were used to account for abundance and evenness of mosquito species present using the formulae below.

$$H=-\sum_{i=1}^{s}P_{i}\;\text{l}\text{n}(P_{i})$$

$$E=\frac{e^{H}}{\text{ln(}S\text{)}}$$

Where H is the Shannon’s diversity index and p _i _is proportion of the species relative to total number of the species. E _H_ is the Shannon’s equitability index calculated by dividing H by the natural logarithm of total number of mosquito species within the community (richness) ^48, 49^. The results were considered significant at p<0.05.

## Results

### Survivorship and species distribution of mosquitoes emerging from eggs collection

**Figure 2.**
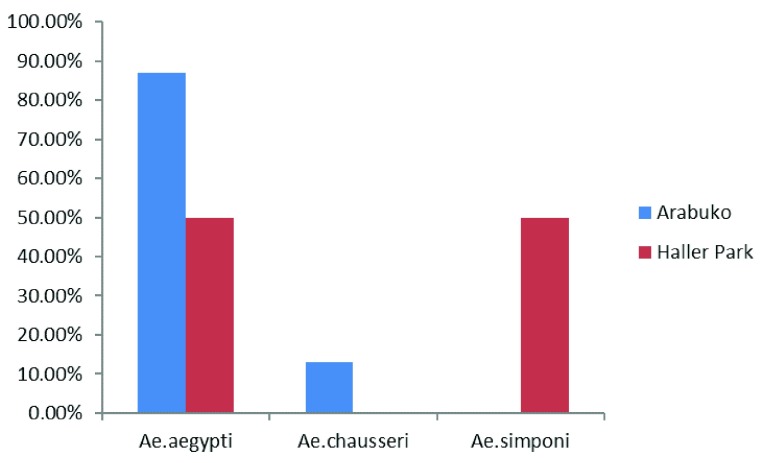
Survivorship and species distribution of mosquitoes emerging from egg collection in forested areas.

Out of 15 ovicups in each of the forested areas, nine were positive for eggs at Haller Park while five were positive in Arabuko-Sokoke forest. Out of 67 eggs that hatched to first instar larvae in the insectary, 60 survived to adulthood. The overall survivorship from L1 to adulthood was 89.5%. The 60 adult mosquitoes belonged to three species in *Aedes* genera: *Ae. aegypti* (78.3%), *Ae. simpsoni* sensu lato (s.l.) (11.7%) and *Ae. chausseri (*10.0%) ^50^. *Ae. aegypti* (87.0%) was the most predominant species in Arabuko-Sokoke forest, with 13.0% belonging to *Ae. chausseri*. *Ae. aegypti* and *Ae. simpsoni* s.l. recorded 50% each in Haller Park ( Figure 2).

### Larval habitat diversity and juvenile mosquito abundance and distribution

A total of 17 artificial habitat types were identified during the study, consisting of tyres, containers, roadside drains, flower axils, house drains, manholes, water troughs, water tanks, ditches, car tracks, flowerpots, swimming pools, puddles, clam shells, fountains and swamps ^50^. There was no significant difference in habitat types and distribution across the study areas (F _(6, 253)_ = 1.46, P < 0.1911). Overall, 260 mosquito larval habitats were identified, the majority being tyres (27%), followed by containers (19%). Other types of habitats sampled included scrap metals, household utensils, abandoned fountains, concrete construction water tanks, dampened polyvinyl chloride (PVC) mat, open water collection area, abandoned trailer, water pipe leakage, and polythene bags. Habitats encountered only once during the study period were classified as other habitats; however, they accounted for 10% of the total habitats sampled across the study area ( Table 1).

**Table 1.** Mosquito juvenile abundance and distribution in different habitats expressed in numbers and percentage of the total collection per site, n (%).

**Sub-county**	**Habitat type**	**Early instars**	**Late instars**	**Pupae**
**Mvita**	Car track (2) ^a^	16(0.67) *	2(0.09)	0(0.00)
	Well (2)	62(2.58)	15(0.71)	2(0.18)
	Road drainage (15)	643(26.80)	468(22.22)	93(8.55)
	House drainage (8)	306(12.76)	344(16.33)	300(27.57)
	Ditch (1)	26(1.08)	81(3.85)	4(0.37)
	Container (12)	354(14.76)	251(11.92)	49(4.50)
	Tyre (23)	589(24.55)	622(29.53)	57(5.24)
	Water tank (2)	98(4.09)	39(1.85)	6(0.55)
	Manhole (3)	190(7.92)	150(7.12)	521(47.89)
	Flower axil (9)	62(2.58)	69(3.28)	34(3.13)
	Other habitats **(6)	53(2.21)	65(3.09)	22(2.02)
	**Total (82)**	**2399(100)**	**2106 (100)**	**1088(100)**
**Changamwe**	Road drainage (7)	173(15.54)	233(18.58)	178(37.16)
	House drainage (2)	43(3.86)	7(0.56)	0(0.00)
	Swamp (1)	7(0.63)	34(2.71)	16(3.34)
	Ditch (1)	5(0.45)	11(0.88)	0(0.00)
	Container (17)	234(21.02)	339(27.03)	112(23.38)
	Tyre (22)	445(39.98)	384(30.62)	77(16.08)
	Water tank (1)	3(0.27)	0(0.00)	0(0.00)
	Water trough (3)	8(0.72)	7(0.56)	3(0.63)
	Flowerpot (1)	5(0.45)	7(0.56)	8(1.67)
	Flower axil (3)	32(2.88)	41(3.27)	78(16.18)
	Other habitats (7)	158(14.20)	191(15.23)	7(1.46)
	**Total (65)**	**1113(100)**	**1254(100)**	**479(100)**
**Kisauni**	Swimming pool (1)	139(11.33)	181(10.73)	0(0.00)
	Car track (2)	5(0.41)	4(0.23)	0(0.00)
	Road drainage (4)	49(4.00)	93(5.51)	44(8.13)
	House drainage (1)	1(0.08)	78(3.91)	95(17.56)
	Puddles (1)	41(3.34)	66(3.91)	46(8.50)
	Ditch (2)	13(1.06)	3(0.18)	1(0.18)
	Container (6)	118(9.62)	151(8.96)	5(0.92)
	Tyre (14)	252(20.55)	310(18.39)	35(6.47)
	Water tank (3)	27(2.20)	35(2.08)	6(1.10)
	Manhole (6)	303(24.71)	270(16.01)	129(23.84)
	Water trough (3)	82(6.69)	88(5.22)	9(1.66)
	Flowerpot (1)	44(3.59)	3(0.18)	0(0.00)
	Flower axil (2)	9(0.73)	19(1.13)	164(30.31)
	Other habitats (13)	143(11.66)	385(22.84)	7(1.29)
	**Total (59)**	**1226**	**1686**	**541**
**Likoni**	Road drainage (1)	32(8.36)	18(5.90)	62(45.59)
	House drainage (2)	49(12.79)	56(18.36)	22(16.18)
	Container (1)	14(3.66)	6(1.98)	2(1.47)
	Tyre (4)	86(22.45)	99(32.46)	24(17.65)
	Water trough (1)	65(16.97)	59(19.34)	0(0.00)
	Flower axil (5)	137(35.77)	67(21.97)	26(19.12)
	**Total (14)**	**383**	**305**	**136**
**Haller Park**	Clam shell (2)	7(17.50)	6(33.33)	6(33.33)
	Tyre (8)	33(82.50)	12(66.67)	12(66.67)
	**Total (10)**	**40**	**18**	**18**
**Bamburi**	Container (8)	18(69.23)	11(73.33)	3(58.18)
	Tyre (5)	8(30.77)	4(26.67)	11(41.82)
	**Total (13)**	**26**	**15**	**14**
**Gede**	Container (5)	17(19.54)	9(21.43)	6(18.18)
	Water tank (3)	70(80.46)	33(78.57)	27(81.82)
	**Total (8)**	**87**	**42**	**33**

^a^The value in parenthesis represents the number of habitats sampled per habitat type. *The values represent the percentages of juveniles per habitat type divided by the total number juveniles in each category per site. ** Other habitats include abandoned fountains, construction cemented tanks, poorly disposed of polyvinyl chloride mats and polythene papers, garbage dumping site, roadside rain water collection, open ornamental pots, poorly disposed of scrap metal and trailers.

A total of 13,009 immature mosquitoes sampled comprised of 10,700 (82.3%) larvae and 2,309 (17.7%) pupae. The larvae were further categorized according to their developmental stage; 5,274 (49.3%) were early instar (L1-L2) larvae, whereas 5,426 (50.7%) late instar (L3-L4). The most productive habitats were tyres, which accounted for 23% of the total immature larvae collected, followed by road drains (16%), containers (13%), manholes (12%) and house drains (10%). Other habitats had a less than 4% juvenile mosquito population, as shown in Table 1. There was significant difference in immature mosquito production among the different types of breeding habitats (F _(16, 243)_ = 3.17, P < 0.0001) across the urban areas of Mombasa Island, Changamwe, Likoni and Nyali. Linear regression analysis showed that water turbidity and the age of the habitats were significant predictors of *Culex* mosquito larval production in an aquatic habitat ( Table 2), with older and highly polluted habitats producing larger *Cx. pipiens* populations.

**Table 2.** Linear regression analysis showing the association between culicine larval population and environmental variables.

Predictor	Beta	P	95.0% Confidence interval for B	
			Lower bound	Upper bound
**(intercept)**		0.006	0.541	3.048
**Predators**	0.064	0.523	-0.090	0.176
**Length**	0.115	0.243	-0.002	0.009
**Width**	-0.171	0.124	-0.198	0.024
**Depth**	-0.016	0.888	-0.224	0.194
**Vegetation (%)**	-0.025	0.799	-0.012	0.009
**Age of habitat**	0.389	0.000	**0.084**	**0.268**
**Land use**	0.089	0.416	-0.092	0.221
**Habitat type**	-0.123	0.262	-0.027	0.007
**Water** **turbidity**	-0.382	0.000	**-0.415**	**-0.126**
**Water flow**	-0.143	0.155	-0.719	0.116

### Species composition and distribution among the different aquatic habitats across the study sites

**Figure 3.**
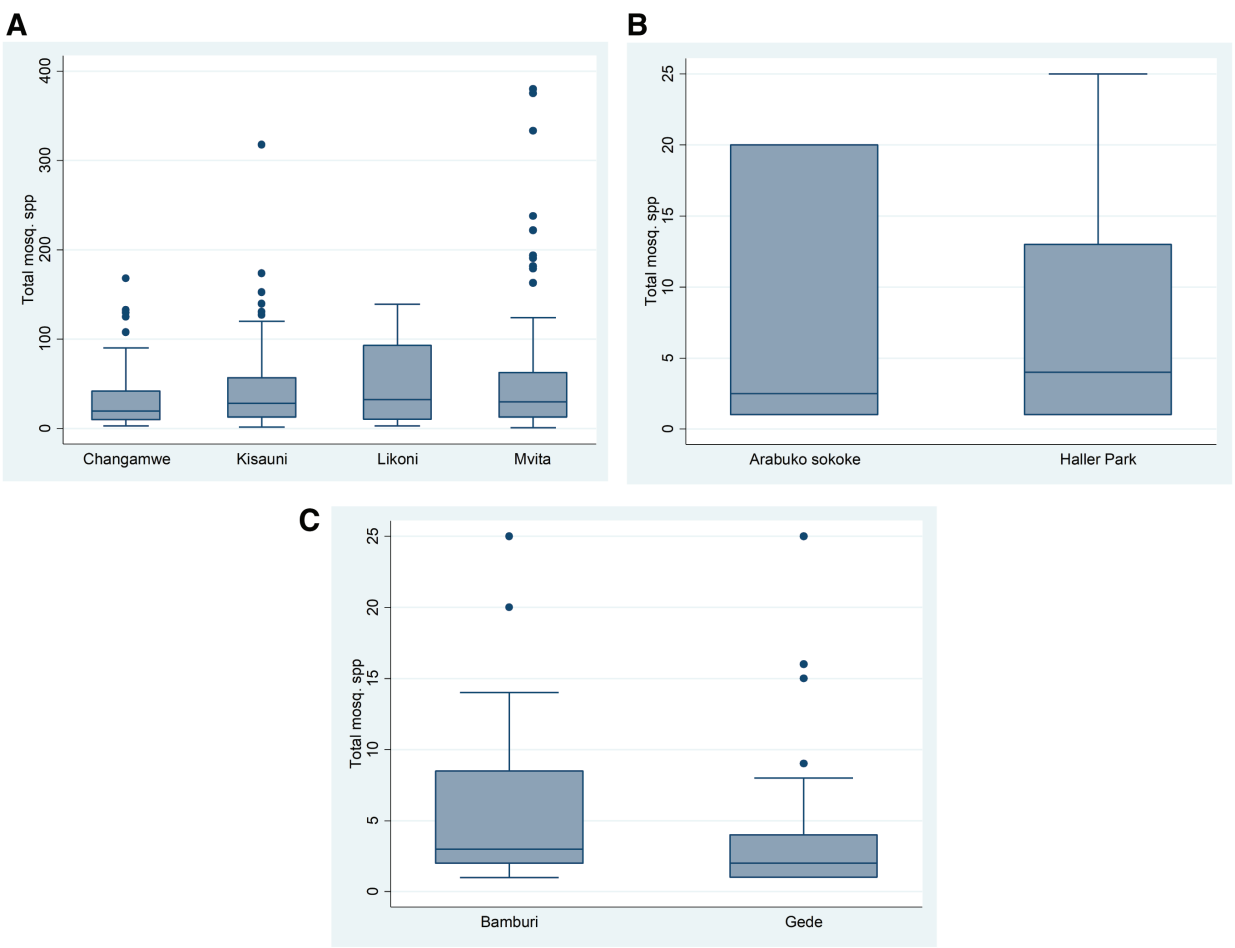
Box plots showing mosquito distribution in different sites within the three ecozones: ( **A**) shows mosquito distribution in different sites across the urban ecozone; ( **B**) mosquito distribution in different sites in the forested areas; and ( **C**) mosquito distribution in peri-urban settings.

**Figure 4.**
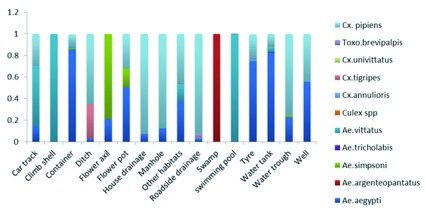
Culicine mosquito species proportions per habitat across all study sites.

The 13,009 mosquito larvae sampled were taxonomically identified as belonging 10 species in three genera, the majority being in *Aedes* (five species) and others in *Culex* (four species) and *Toxorhynchites* (one species). *Aedes aegypti* and *Culex pipiens* were the most dominant species among the larval samples collected during the study period. Among the 10 species identified, *Cx. pipiens* (49%) ** was the highest, followed by *Ae. aegypti* (39%), **Ae. vittatus (6%), Ae. simpsoni s.l. (4%), Cx. tigripes* and **Tx. brevipalpis* (1% each), with Ae. argenteopantatus, Ae. tricholabis, Cx. annulioris* and Cx. univittatus* recording less than 1% each ( Figure 3). Only *Ae. aegypti* was identified at all the study sites. Changamwe recorded the highest number of mosquito species (eight species), followed by Gede (six species), Likoni and Mombasa Island (five species each) and Haller Park and Bamburi (two species each) ( Figure 4).

In the Mombasa Island area, the most predominant species were *Cx*. **pipiens* (59%) and *Ae. aegypti* (37%). The most predominant species in other areas were: *Ae. aegypti* (54%) and *Cx. pipiens* (31%) in Changamwe; *Cx. pipiens* (50%), Ae. aegypti* (35%) and *Ae. vittatus* (14%) in Kisauni; *Cx*. *pipiens* (57%) and *Ae.simpsoni s.l.* (28%) in Likoni; *Ae. vittatus* (89%) and *Ae. aegypti* (11%) in Haller Park; *Ae. aegypti* (78%) and *Cx. pipiens* (19%) in Gede; and *Ae. aegypti* (87%) and *Cx. pipiens* (13%) in Bamburi. The rest of the species recorded less than 5% each in each of the above study sites. *Ae. aegypti* was the dominant species in containers, water tanks, tyres and flowerpots. Only *Ae. aegypti* species were sampled along the beach line of the Indian Ocean during the study period. *Cx. pipiens* was the most predominant in ditches, house drains, manholes, road drains and water troughs and no mosquitoes of this species were sampled from swamps, flower axils and swimming pools, with *Ae. simpsoni* s.l. dominating in samples from the flower axils.

*Ae. aegypti* was the most predominant species in peri-urban (80.2%) areas and second most predominant in urban (38.7%) areas, while *Cx. pipiens* mosquitoes were the most predominant in urban areas (50.2%) and second most predominant in peri-urban (17.1%). *Ae. vittatus* (86.0%) were the most predominant in forest eco-zones, followed by *Ae. aegypti* (10.5%). Tyres were the most productive larval habitat in forested and urban areas, while water tanks were the most productive larval habitat in peri-urban areas, as shown in Table 3.

**Table 3.** Mosquito species distribution in different habitats expressed in numbers (n) and percentages (%) of the total collection per eco-zone.

Eco-zones	*Ae. Aegypti*	*Ae.* *argenteopantatus*	*Ae.* *simpsoni*	*Ae.* *tricholabis*	*Ae. vittatus*	*Cx. pipiens*	*Cx.* *annulioris*	*Cx.* *tigripes*	*Cx.* *univittatus*	*Tx.* *brevipalpis*	Grand total
**Forest**	8(10.5) *	**0**	**0**	**0**	**68(89.0)**	**0**	**0**	**0**	**0**	**0**	**76**
Clam shell	0	0	0	0	19(100.0)	0	0	0	0	0	19(25.0)
Tyre	8(14.0)	0	0	0	49(86.0)	0	0	0	0	0	57(75.0)
**Peri-urban**	**174(80.2)**	**0**	**1(0.5)**	**2(0.9)**	**0**	**37(17.1)**	**1(0.5)**	**0**	**1(0.5)**	**0**	**216**
Container	57(89.1)	0	1(1.6)	0	0	4(6.3)	1(1.6)	0	1(1.6)	0	64(29.5)
Tyre	17(73.9)	0	0	0	0	6(26.1)	0	0	0	0	23(10.6)
Water tank	100(76.9)	0	0	2(1.5)	0	2720.8)	0	0	0	0	130(59.9)
**Urban**	**4925(38.7)**	**57(0.5)**	**480(3.8)**	**0**	**662(5.2)**	**6379(50.2)**	**16(0.1)**	**92(0.7)**	**0**	**105(0.8)**	**12716**
Car track	4(14.8)	0	0	0	15(55.6)	8(29.6)	0	0	0	0	27(0.2)
Container	1397(85.4)	0	17(1.0)	0	36(2.2)	161(9.9)	16(1.0)	0	0	8(0.5)	1635(12.9)
Ditch	4(2.8)	0	0	0	0	94(65.3)	0	46(31.9)	0	0	144(1.1)
Flower axil	113(21.0)	0	425(76.0)	0	0	0	0	0	0	0	538(4.2)
Flower pot	80(50.6)	0	27(17.1)	0	0	51(32.3)	0	0	0	0	158(1.2)
House drainage	93(7.2)	0	0	0	0	1205(92.6)	0	3(0.2)	0	0	1301(10.2)
Manhole	207(12.1)	0	0	0	25(1.5)	1484(86.5)	0	0	0	0	1716(13.5)
Other habitats	474(37.5)	0	0	0	202(16.0)	578(45.8)	0	6(0.5)	0	3(0.2)	1263(9.9)
Roadside drainage	54(2.6)	0	0	0	20(1.0)	1938(92.9)	0	23(1.1)	0	51(2.4)	2086(16.4)
Swamp	0	57(100.0)	0	0	0	0	0	0	0	0	57(0.5)
swimming pool	2(0.6)	0	0	0	318(99.4)	0	0	0	0	0	320(2.5)
Tyre	2217(75.2)	0	7(0.2)	0	27(0.9)	640(21.7)	0	14(0.5)	0	43(1.5)	2948(23.2)
Water tank	184(86.0)	0	0	0	19(8.9)	11(5.1)	0	0	0	0	241(1.7)
Water trough	52(22.6)	0	4(1.7)	0	0	174(75.7)	0	0	0	0	230(1.8)
Well	44(55.7)	0	0	0	0	35(44.3)	0	0	0	0	79(0.6)
**Grand Total**	**5107(39.3) ^a^**	**57(0.4)**	**481(3.7)**	**2(0.0)**	**730(5.6)**	**6416(49.3)**	**17(0.1)**	**92(0.7)**	**1(0.0)**	**105(0.8)**	**13008**

*Mosquito species calculated in percentages per habitat within an eco-zone; ^a^ percentages of mosquito species calculated per species across the three eco-zones.

Mosquito species distribution appears to be diversely within Changamwe compared to the other three urban areas and lower in Arabuko-Sokoke forest compared to the Haller Park forested area. Species distribution also appears to be lower in Gede compared to Bamburi, where it appears to be diverse, as shown in the box plot in Figure 3.

### Adult species diversity and distribution

A total of the 4675 adult mosquitoes belonging to 18 species in six genera were collected from the four adult sampling sites of Haller Park, Bamburi, Arabuko-Sokoke forest and Gede. Among the six genera, the majority belonged to the *Aedes* genus (eight species), followed by *Culex* (five species), *Anopheles* (two species) and *Mansonia* (two species), while one species belonged to *Eretmapodite* and *Ficabia* each. Overall, *Ae. tricholabis (49%)* was the most common species in these collections, followed by *Ae. aegypti* (17%), An. funestus (15%), *Cx. pipiens* (6%), *Cx. vansomereni* (5%), *Cx. univittatus* (3%) and *Ae. vittatus* (1%). Other species including *Ae. simpsoni s.l., Er. chrysogaster, Ma. africanus, Ma. uniformis, An. coustani, Ae. mcintoshi, Ae. hirsutus, Ae. tarsalis, Cx. annulioris, Cx. tigripes, Ficalbia mediolineata* and unidentified species recorded less than 2% each ( Figure 3).

**Figure 5.**
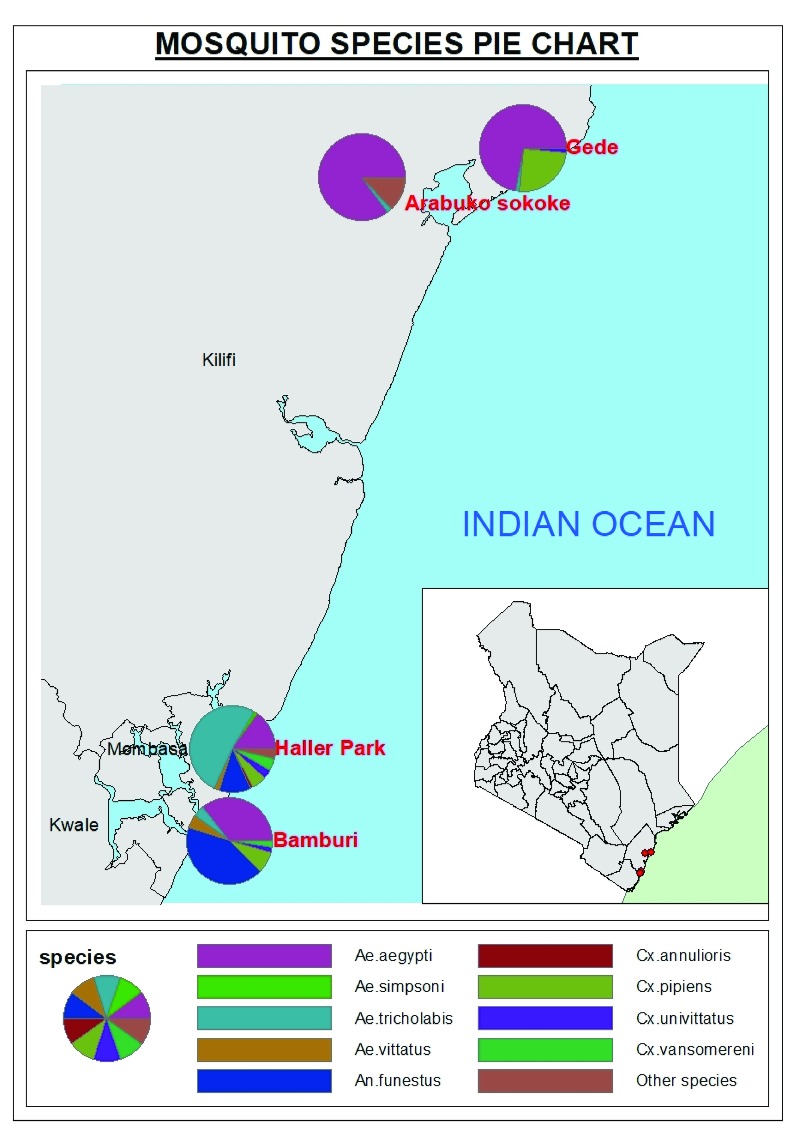
Map showing mosquito species diversity and distribution in peri-urban and forested areas. Other species include *Ae.hirsutus, Ae.chausseri, Ae.mcintoshi, Ae.tarsalis, An.caustani, Cx.tigripes, Er.chrysogaster, Fi.mediolineata, Mn.uniformis* and *Ma.africanus*.

Haller Park recorded the highest number of mosquitoes caught (87.2%), followed by Bamburi (7.9%), Gede (4.0%) and Arabuko-Sokoke forest (0.9%). *Ae. tricholabis* was the only species distributed across the four study sites, as shown in Figure 3. Haller Park recoded the highest number of species (18), Bamburi and Gede recorded 10 and five species, respectively, while Arabuko Sokoke recorded the least with one species. *Ae. tricholabis* (52%) was the dominant species in Haller Park, *An. funestus* (51%) in Bamburi, while *Cx. pipiens* was dominant in Gede (48%), as shown in Figure 5.

### Species diversity and evenness in Mombasa Island and its environs

Shannon diversity index showed that mosquito species diversity (H) and evenness (E _H_) was highly significant in Changamwe (H=1.208, E _H_=0.581) for juvenile mosquito species compared to the other sites and Haller Park (H=1.571, E _H_=0.544) for adult mosquito species. Arabuko-Sokoke forest recorded the lowest species diversity (H=0.482, E _H_= 0.439). This shows that there was a larger number of mosquito species in Changamwe (eight) and in Haller Park (18) and the individuals within these communities were more equitably distributed among these species, as shown in Table 4.

**Table 4.** Mosquito species diversity and evenness across the study sites.

	Site	*Aedes* **species**	Non- *Aedes* mosquito species	All mosquito species
**Shannon's diversity index (H)**	Changamwe	0.548(4) *	0.549(4)	1.208(8)
	Likoni	0.728(3)	0.038(2)	1.014(5)
	Mvita	0.325(3)	0.073(2)	0.849(5)
	Nyali/kisauni	0.740(3)	0.023(2)	1.068(5)
	Haller Park	0.729(7)	1.581(11)	1.571(18)
	Gede	0.141(3)	0.397(4)	0.793(7)
	Bamburi	0.684(3)	0.835(7)	1.456(10)
	Arabuko Sokoke	0.482(3)	0.000(0)	0.482(3)
**Shannon's equitability (EH)**	Changamwe	0.396(4)	0.396(4)	0.581(8)
	Likoni	0.663(3)	0.055(2)	0.630(5)
	Mvita	0.296(3)	0.105(2)	0.527(5)
	Nyali/kisauni	0.674(3)	0.033(2)	0.664(5)
	Haller Park	0.374(7)	0.659(11)	0.544(18)
	Gede	0.128(3)	0.286(4)	0.408(7)
	Bamburi	0.632(3)	0.429(7)	0.632(10)
	Arabuko Sokoke	0.439(3)	0.000(0)	0.439(3)

*The values in parenthesis represent the number of species collected in individual study site.

## Discussion

 Knowledge on larval habitat diversity in an area and their influence on mosquito species diversity, abundance and distribution is important in informing integrated vector control strategies and mitigation of future disease outbreak ^2^. These habitats include mangrove forests, forests, woodlands, flood plains, swamps, urban and peri-urban areas. The majority of breeding sites in these habitats, especially in urban and peri-urban areas, arise from human or animal activities such as footprints, car tracks, puddles, hoof prints, containers, tyres, house drains chambers and other artificial aquatic habitats ^2, 51^.

This study collected 13,009 juvenile mosquitoes from 17 diverse mosquito larval habitats within the study sites. Among these habitats, tyres, containers, road drains, manholes and house drains were the most productive for all immature stages of mosquitoes. Tyres were found to be an important habitat in Mombasa Island, Changamwe, Nyali, Likoni and Haller Park, especially for the *Ae. aegypti* larval production. This agrees with previous studies conducted in Mombasa and Malindi that found tyres and containers to be important habitats for immature *Ae. aegypti* productivity in urban setting ^13, 52^. The high number of tyres in urban areas was due to poor disposal mechanisms of old tyres, while in Haller Park this was due to large piles of old tyres collected for use as an alternative source of energy in the Bamburi cement plant. Urban areas in developing countries like Kenya regularly experience water shortages, especially in the dry season. Consequently, residents are forced to store water in basins, small tanks and jerry cans. These containers provide breeding sites for *Ae. aegypti* during the dry season, increasing their densities, which has often been associated with dengue and chikungunya outbreaks during the dry season ^13, 53^.

Blocked and poorly maintained manholes, road drains and house drains played a significant role in *Cx. pipiens* production, a vector of filarial worms and WNV and a secondary vector of RVFV ^1, 2, 9^. Plant axils were found to be important breeding sites for *Ae.simpsoni* in Likoni, mainly on axils of *Colocasia esculenta* and *Canna edulis* potted plants in government, business and residential premises. Habitats encountered once and classified under others in urban areas also played significant role in larval and pupae production, with *Cx. Pipiens* (46%), *Ae. aegypti* (38%) and *Ae. vittatus* (16%) the most predominant species in these habitats. These habitats include abandoned fountains, construction cemented tanks, poorly discarded PVC mats and polythene papers, garbage dumping sites, roadside rainwater collections, open ornamental pots, poorly discarded scrap metal and trailers in garages, among others. All habitats were found to contain high numbers of late instar larvae and pupae, indicating their ability to attract gravid culicine mosquitoes for oviposition and successfully support the development of the immature mosquito to adult stages. Hence, owing to their productivity and stability, these habitats should be the primary target for vector control in the region.

 Aggregated distribution of culicine immature stages observed within different larval habitats indicates that the dynamic interaction of factors in different aquatic habitats such as nutrients, social interactions and physical features influences the diversity and distribution patterns of immature mosquitoes ^28^. Water turbidity and age of habitats were found to be important environmental variables in determining the abundance and diversity of culicine mosquito larvae. Previous studies in Mwea also demonstrated a positive correlation between water turbidity and *Culex* mosquito larvae production ^54, 55^. *Culex* larvae were found to colonize aquatic habitats polluted with sand, mud, sewage and garbage more than *Aedes* species, the majority of which were found to colonize fairly clean, unpolluted water. A similar observation was reported in other studies, which found that organically polluted water favoured the breeding of *Cx. pipiens* larvae ^2, 28^. This is an indication that the water produces chemical cues that attract gravid culicine mosquitoes to lay eggs and the organic polluted water is rich in nutrients for the successful development of the *Culex* mosquito immatures.

 This study found 18 mosquito species, which have been described in different previous studies, along the coast of Kenya based on mosquito larvae and adult sampling ^13, 56, 57^. The majority of these species were found to co-exist in diverse habitat types, except *Ae. Argenteopantaus,* which was found to occur singly in the swamp. This indicates that mosquito species share food resources within these habitats and hence, ensures continuous production of adult mosquitoes throughout the year. *Ae. aegypti* larvae were distributed in diverse habitat types but were most predominant in tyres, water tanks, flowerpots, containers and wells, and were less predominant in swimming pools, with none in swamps or clam shell ^58^. The current study found more diversity in *Ae. aegypti* larvae habitats compared to the previous study, where *Ae. aegypti* was described mainly as breeding in containers and tyres ^13, 52, 57^. *Ae. aegypti* mosquitoes were also found to co-exist with the other species sampled in different eco-zones. Other *Aedes* species were also found to breed in wide range of aquatic habitats, although they occurred in smaller numbers as compared to *Ae. aegypti*. *Ae. Simpsoni* s.l *.*, an axillary breeding mosquito, predominantly occurred in flower axils, an observation that had been made previously in a larval surveillance study in Tanzania ^58^. Small numbers of *Ae.simpsoni* s.l. were found to occur in water troughs, tyres, containers and flowerpots in decreasing order of abundance. The highest population density of *Ae. vittatus* occurred in an abandoned swimming pool, where they were the most predominant species. Predominance of *Ae. vittatus* (89%) in the forested zone is a great risk factor for arbovirus outbreak given its role in the maintenance and transmission of arboviruses such as CHIKV, ZIKV and DENV ^5, 11, 59– 61^. The occurrence of *Ae. aegypti* in ocean water-filled tyres, wells and containers sampled along the beach line of the Indian Ocean is an indication that they are able to tolerate high salinity levels in their aquatic habitat compared to other species. This was also observed in a laboratory study, which found that coastal *Ae. aegypti* is more adaptive as compared to plateau populations ^62^. This study found *Ae. aegypti* to be the most predominant species in peri-urban areas (80.2%) and the second most predominant species in urban areas (38.7%). This poses a great risk of arbovirus outbreaks in the event of a spill-over from the sylvatic cycle to the peri-urban area, with *Ae. aegypti* being the main vector for urban amplification and transmission of DENV, CHIKV, ZIKV and YFV along the Kenyan coast ^3, 5, 10, 13^.

*Cx. pipiens* was the most predominant *Culex* species sampled in the urban areas. This is in agreement with other studies in urban Malindi and Mombasa, which showed that *Cx. quinquefaciatus*, a member of *Cx. pipiens* complex, was the most predominant *Culex* species in urban Malindi and Mombasa Island ^13, 63^ They predominantly breed in roadside drains, manholes and household drains. In all habitats, they were found to co-exist with other species. There was no *Culex* species in flower axils, swimming pools, clam shells and swamps. The other three *Culex* species occurred in very small numbers across diverse aquatic habitats. The existence of different mosquito species in diverse aquatic habitats demonstrates their adaptation to those habitats. This poses a great risk to the control of these mosquito species and hence, risk of mosquito-borne infection outbreaks, given that high vector densities are associated with vector-borne disease outbreaks ^64^.

 The significant disparity observed in mosquito species diversity and richness across the study sites is due to diversity in mosquito breeding habitats. Changamwe had diverse larval habitat types, which supported diverse species production. The high species diversity and evenness in distribution in Haller Park during this study period, despite the absence of short rains, shows that the water pools, fishponds and high number of discarded tyres play a significant role as breeding habitats. Low species diversity in Arabuko-Sokoke forest could be explained by the dry weather experienced during this period along the Kenyan coast, due to no short rains. Although there were no larvae sampled from tree holes, rock holes and plant axils in forested areas, a number of eggs belonging to *Ae. chausseri, Ae. aegypti* and *Ae. simpsoni* s.l. were collected in ovicups placed on tree holes, rock holes and between branches within the two forests. This shows that in the event that there was water collection in those locations due to rain, they would play a significant role as breeding sites for these three species and others that were not sampled in the current study. The high number of *Aedes* species diversely distributed along the Kenyan coast poses a significant risk of arbovirus outbreak in urban and peri-urban areas in Mombasa and Kilifi counties. This could explain the dengue and chikungunya outbreaks reported in Mombasa county in recent years ^19, 24, 53^.

Human behaviour and socioeconomic settings play significant roles in larval habitat generation and, consequently, high larval production in the study areas. The presence of a large adult mosquito population indicates the availability and ability of the habitats to support juvenile populations to adulthood. This demonstrates that there is significant risk of mosquito-borne arbovirus, such as DENV, ZIKV, YFV and CHIKV, and parasitic filarial worm infection outbreaks along the Kenyan coast. Successful integrated vector control (IVC) involves control strategies that target both mosquito larvae and adults. Targeted larval source management strategies should be implemented by the county health team, targeting diverse aquatic habitat types in each individual eco-zone, with the most productive aquatic habitats given priority in the fight against mosquito-borne infections. Given the majority of these mosquito breeding habitats are man-made, creating awareness at all levels would be an effective tool in reducing larval habitats. Proper tyre, container and other waste disposal mechanisms and installing and maintaining drainage systems would reduce mosquito populations in urban and peri-urban areas. This would call for door-to-door campaigns, as majority of these mosquito breeding habitats were found in commercial and residential properties. Ensuring a regular tap water supply to avoid water storage containers will reduce the container-breeding *Ae. aegypti* population and, consecutively, drought-associated arbovirus outbreaks in the region. Proper larval management and adult mosquito interaction prevention strategies should be effectively employed, especially by forest-neighbouring dwellers to prevent sylvatic transmission spill-over to peri-urban, which could initiate urban outbreaks. Further regular surveillance for both juvenile and adult mosquitoes in urban and forested areas along the coastal line will help to describe the composition of all mosquito species within these areas and establish the magnitude of vector-borne diseases.

## Data availability

Figshare: Larval habitat diversity and mosquito species distribution along the Coastal Kenya.

https://doi.org/10.6084/m9.figshare.10073099 ^50^.

The project contains the following underlying data:

- Mosquito Larval habitats diversity, distribution, characterization and species diversity.xlsx
- Mosquito species distribution along the Coastal Kenya.xlsx

Data are available under the terms of the Creative Commons Zero "No rights reserved" data waiver (CC0 1.0 Public domain dedication).
