# Supplementary material for: Larval habitat diversity and mosquito species distribution along the coast of Kenya
Source: Wellcome Open Res. 2019 Nov 13;4:175. [Version 1] doi: 10.12688/wellcomeopenres.15550.1 (PMC7241275; doi:10.12688/wellcomeopenres.15550.1)
Supplement: Peer review history

# Peer review: Reviewer response for version 1

**DOI:** 10.21956/wellcomeopenres.17027.r37047

**Published:** 2020-05-20

**Authors:** Francis M. Mutuku

**Affiliations:** 1 Department of Environment and Health Sciences, Technical University of Mombasa, Mombasa, Kenya

**General comments:**

- The manuscript "Larval habitat diversity and mosquito species distribution along the coast of Kenya" provides insightful information on mosquito diversity at both immature and adult stages. The knowledge gained can be utilized in vector control and surveillance of mosquito-borne diseases.

**Abstract:**

- Total species reported in the results is 18 but 19 in the abstract.

**Introduction:**

- "Potential 'spill-over' effect can easily be detected if the existing mosquito species in both forest and urban ecologies are known". This is a suggested addition in the introduction section as justification for this study.

**Methods:**

- Selection of study was well thought and representative of the different types of mosquito ecology in the study areas and the mosquito collection methods were appropriate.
- There is repetition in the description of the study area and site selection.
- Why were *Aedes* mosquito eggs not collected in urban and peri-urban areas? It is not mentioned under egg collection. How many days were the ovicups left out in the field? Were there replicates in egg collection?
- Describe the sampling procedures used to identify the sampled larval habitats within each of the ecological zones. For example, in Changamwe; did you sample in open areas or inside or outside houses? What was the coverage of the sampling within each ecological zone? What was the sampling unit for larval habitat sampling? All these details are required.
- Adult sampling was biased against day-feeding mosquitoes. This should appear as a limitation of the study.
- Mosquito count data is usually over-dispersed thus rendering ANOVA an inappropriate statistical test. It is recommended that the authors ascertain if ANOVA was the correct statistical test for the count data.

**Results:**

- It is not indicated if there were larval habitats with immature mosquitoes in Arabuko-Sokoke.
- Immature species data was not provided in the immature data file. It is surprising that there were no Anophelines identified from the immatures. 
- Figure 3: it is not clear how figure 3 combines mosquito diversity for both adults and immatures. 
- Results of the Linear regressions analysis to test the relationship between the culicine larval population and environmental variables are not presented in here.

**Discussion:**

- There is a lot results in the discussion. The discussion themes are mixed-up. Re-identify the main discussion themes and make the discussion more focused.
- The following references may enrich your discussion:

1. Forsyth *et al.* (2020 ^1^).
2. Ndenga *et al.* (2017 ^2^).
3. Ngugi *et al.* (2017 ^3^). 

- A limitation paragraph is required in the discussion.

**Conclusions:**

- The introduction is fairly well written. However, all the other sections of the manuscript need to be rewritten carefully as per the comments above. 

Is the work clearly and accurately presented and does it cite the current literature?

Yes

If applicable, is the statistical analysis and its interpretation appropriate?

Partly

Are all the source data underlying the results available to ensure full reproducibility?

Partly

Is the study design appropriate and is the work technically sound?

Yes

Are the conclusions drawn adequately supported by the results?

Yes

Are sufficient details of methods and analysis provided to allow replication by others?

Partly

Reviewer Expertise:

Public health entomology and epidemiology

I confirm that I have read this submission and believe that I have an appropriate level of expertise to confirm that it is of an acceptable scientific standard.

## References

[1] : Source reduction with a purpose: Mosquito ecology and community perspectives offer insights for improving household mosquito management in coastal Kenya. *PLoS Negl Trop Dis*.2020;14(5) : 10.1371/journal.pntd.0008239 e0008239 10.1371/journal.pntd.0008239 32392226PMC7241847

[2] : Characteristics of Aedes aegypti adult mosquitoes in rural and urban areas of western and coastal Kenya. *PLoS One*.2017;12(12) : 10.1371/journal.pone.0189971 e0189971 10.1371/journal.pone.0189971 29261766PMC5736227

[3] : Characterization and productivity profiles of Aedes aegypti (L.) breeding habitats across rural and urban landscapes in western and coastal Kenya. *Parasit Vectors*.2017;10(1) : 10.1186/s13071-017-2271-9 331 10.1186/s13071-017-2271-9 28701194PMC5508769

# Peer review: Reviewer response for version 1

**DOI:** 10.21956/wellcomeopenres.17027.r37794

**Published:** 2020-03-09

**Authors:** John Paul Mutebi

**Affiliations:** 1 Centers for Disease Control and Prevention (CDC), Fort Collins, CO, USA

**General comments:**

- The MS "Larval habitat diversity and mosquito species distribution along the coast of Kenya" investigated mosquito larval habitats and adult mosquito adult species diversity in locations along the Kenyan coast. The information gained is important and interesting. The data analysis was exhaustive and the write up is understandable. 
- However, the narrative is too wordy. There are numerous repetitions of words and phrases all over the MS. The aims were not clearly outlined and it is difficult to see the connection between the introduction and the discussion. The MS needs some rewriting to improve clarity.
- This starts with the introduction "Larval habitat diversity and mosquito species distribution along the coast of Kenya". I think this should be "Mosquito larval habitats and species diversity on the Kenyan coast". 

**Abstract: **I think the abstract should be improved to:

**"Background:** With a few exceptions, prevention and control of arboviral diseases depend solely on the control of the vectors. Effective vector control measures are guided by entomological parameters such as vector species distribution and relative abundance and these parameters are monitored through routine vector surveillance. The current study investigated mosquito larval habitats, mosquito species distribution and diversity in selected sites along the coast of Kenya.

**Methods:** Larval and adult mosquito sampling were conducted in different ecological zones in Mombasa and Kilifi counties. Larval surveys were conducted using dippers and turkey basters (large pipettes) and adult sampling was by using BG-Sentinel Traps, CO2 Baited Light Traps and CDC resting traps.

**Results:** We collected 13,009 larvae and pupae from 17 diverse aquatic habitats. Larval productivity differed significantly (F _(16, 243)_ = 3.21, P < 0.0001) among the habitats; the highest number of larvae and pupae were collected from discarded tyres. *Culex pipiens *(50.17%) and *Aedes aegypti* (38.73%) were the most abundant species in the urban areas, and *Ae. vittatus* (89%) was the most abundant species in forested areas.  Overall, 4,735 adult mosquitoes belonging to 19 species were collected.  Higher densities of *Ae. aegypti* were detected in urban areas compared to the peri-urban and  the forests, However, forests supported greater mosquito species diversity.

**Conclusions:** High *Ae. aegypti* production in urban and peri-urban areas present a risk of arbovirus transmission. Targeting *Ae. aegypti *larval habitats especially discarded tyres, discarded artificial containers and poorly maintained drainage systems could have a significant impact on *Ae. aegypti* population sizes and arboviral transmission along the coast of Kenya."

**Introduction:**

- I think the first two paragraphs should be omitted
- Beginning with the third paragraph:

"Kenya has a history of arboviral disease outbreaks such as yellow fever (YF), Rift Valley fever (RVF) dengue (DEN)and chikungunya (CHIK) ^17– 22^.  In 2013 and 2014, DEN outbreaks occurred in Mombasa and its neighborhoods and during these outbreaks more than 100 laboratory confirmed cases were detected. The majority of the cases were the elderly and the children ^13, 19, 23^. Recently, in 2017 and 2018, DEN and CHIK outbreaks were reported in Mombasa, along the Kenyan coast, and in Mandera in northeastern Kenya ^24– 26^."

 I am afraid I cannot rewrite the entire MS, but I think the authors now have an idea of how to improve the narrative and the clarity of the MS. I am going to stop here and leave it to the authors.

 One more thing, the authors repeatedly used *Cx. pipiens* in the narrative. Is that *Cx. pipiens* or *Cx. quinquefasciatus*? How were they able to differentiate the two species?

Is the work clearly and accurately presented and does it cite the current literature?

Partly

If applicable, is the statistical analysis and its interpretation appropriate?

Yes

Are all the source data underlying the results available to ensure full reproducibility?

Yes

Is the study design appropriate and is the work technically sound?

Partly

Are the conclusions drawn adequately supported by the results?

Partly

Are sufficient details of methods and analysis provided to allow replication by others?

Yes

Reviewer Expertise:

Medical Entomology, arbovirology, Vector Biology,

I confirm that I have read this submission and believe that I have an appropriate level of expertise to confirm that it is of an acceptable scientific standard, however I have significant reservations, as outlined above.

# Peer review: Reviewer response for version 1

**DOI:** 10.21956/wellcomeopenres.17027.r37741

**Published:** 2020-02-18

**Authors:** Bryson Alberto Ndenga

**Affiliations:** 1 Centre for Global Health Research, Kenya Medical Research Institute, Kisumu, Kenya

**Summary:**

 This research study was designed and conducted to describe the diversity of mosquito larval habitats and the distribution of mosquito species in Mombasa and Kilifi Counties along the Kenyan coast. The sites were categorized as two forested areas (Arabuko Sokoke forest and Haller Park), two peri-urban areas (Gede and Bamburi) and urban areas (Changamwe, Mombasa Island, Nyali and Likoni). Production of *Aedes aegypti* was found to be high in urban and peri-urban areas which presents a great risk of arbovirus outbreaks.

**Review comments:**

1. Pages 3, 6 and 14: Ensure that there is a space between generic and species parts of the scientific names and that the species name starts with a small letter.
2. Page 4: Figure 1 – make the map of Kenya to be more visibly clear.
3. This paper is about “Larval habitat diversity and mosquito species distribution along the coast of Kenya”, which means all mosquito types should have been targeted during the sampling period. However, there is a tendency of some bias towards sampling of Culicines and more specifically *Aedes* mosquitoes as opposed to the Anophelines. This is clearly indicated by the methods that were used to sample mosquito eggs and adult mosquitoes. Furthermore, it is not indicated whether any sampling was done inside houses (indoors), where other mosquitoes like *Anopheles gambiae s.l*. would have been collected using other methods like pyrethrum spray collections.
4. Page 5: The method you used to determine the age of habitats seems to have been very much unreliable.
5. Page 5: Did you consider the time of the day while determining whether a habitat was partially or fully shaded?
6. Page 5: The way you defined the vegetation cover as “none, some or many plants/grasses” appears relative. How did you ensure this was standardized especially if it was done by several people? Use of percentage coverage can be better.
7. Page 6: Figure 2 – remove the two decimal places on the vertical axis and have species names in italics.
8. Pages 8 and 9: Table 1 – separate the Early and Late instars larvae of Anophelines and Culicines.
9. Page 10: Merge A, B and C into one figure with the same vertical scale for easy comparison.
10. Page 12: Figure 5 – only show the mosquito species pie charts and the descriptions below. Omit the study site map in the background since you already have it in Figure 1.
11. Pages 13 and 14: Plant axils and the “The occurrence of *Ae. aegypti* in ocean water-filled …” have not been indicated anywhere under the Results section, but you are discussing them. This is not in order.
12. Pages 14: If you have to, start sentences with full species name like *Culex pipiens* instead of *Cx. pipiens*.
13. Page 15: Include a paragraph on the limitations of this study and another paragraph on the conclusion.

Is the work clearly and accurately presented and does it cite the current literature?

Yes

If applicable, is the statistical analysis and its interpretation appropriate?

I cannot comment. A qualified statistician is required.

Are all the source data underlying the results available to ensure full reproducibility?

Yes

Is the study design appropriate and is the work technically sound?

Partly

Are the conclusions drawn adequately supported by the results?

Yes

Are sufficient details of methods and analysis provided to allow replication by others?

Partly

Reviewer Expertise:

Medical Entomologist with vast experience in the ecology and control of mosquitoes.

I confirm that I have read this submission and believe that I have an appropriate level of expertise to confirm that it is of an acceptable scientific standard, however I have significant reservations, as outlined above.
